# The prevalence of dental agenesis among children with cleft lip and palate patients in Lahore, Pakistan

**DOI:** 10.12669/pjms.40.3.7997

**Published:** 2024

**Authors:** Naauman Zaheer, Maliha Shabaz, Usman Zaheer, Amjad H. Wyne

**Affiliations:** 1 Naauman Zaheer, BDS, MFDS RCSEd, PhD, CHPE Associate Professor, Oral Biology Department CMH Lahore Medical College & Institute of Dentistry, National University of Medical Sciences, Lahore, Pakistan; 2 Maliha Shabaz, M.Phil,CHPE Assistant Professor, Oral Biology Department, Department of Restorative Dentistry, Faculty of Dentistry, University of Malaya, Kuala Lumpur, 50603, Malaysia; 3 Usman Zaheer, FCPS, M Orth RCSEd (UK), CHPE Associate Professor, Orthodontics Department, Lahore Medical & Dental College, Lahore, Pakistan; 4 Amjad H. Wyne, MDS, Dr. Med. Dent, CHPE Professor, Pediatric Dentistry Department, Pakistan Academy of Pediatric Dentistry, Lahore, Pakistan

**Keywords:** Bilateral cleft (CLPB), Cleft lip and palate (CLP), Cone bean computed tomography (CBCT), Hypodontia, Midline cleft (CLPM), Prevalence, Tooth agenesis

## Abstract

**Objective:**

This retrospective, cross-sectional analytical study investigated the incidence of tooth agenesis in cleft lip and palate (CLP) patients. Cone Beam Computed Tomography (CBCT) radiographs of the CLP children were examined for congenitally missing teeth.

**Method:**

This study was conducted at three radiology centers in Lahore, namely, the Pakistan Jinnah MRI and Body Scan Centre, the University of Lahore Radiology Centres, and Fatima Memorial Hospital, from September 2021 to August 2022. The CLP patients were divided into four groups based on the location of the cleft: Cleft Lip and Palate Right (CLPR), Cleft Lip and Palate Left (CLPL), Bilateral Cleft (CLPB), and Midline Cleft (CLPM), inside and outside the cleft region. Two-way ANOVA was employed to compare the means of agenesis. Tukey’s test was utilized to ascertain where the difference lies. The significance level was set at *p* ≤ 0.05.

**Results:**

Moreover, a significant number of missing teeth were found inside the cleft. This study observed the CLPL (42.3%) and CLPR (13.6%) types more in number. Maxillary first premolars were found more missing outside the cleft region in CLPL and CLPB types. Although CLPB and CLPM types revealed a pattern of missing teeth, only a few cases were found in this study. Moreover, mean tooth agenesis was highest (4.5 SD.71) in the CLPM group, followed up by CLPB (2.75 SD 2.49), CLPR (1.23 SD 1.27), and CLPL Group (1.15 SD 1.12).

**Conclusions:**

Unilateral cleft lip and palate patients reported significant agenesis patttern compared to bilateral and median cleft cases.

## INTRODUCTION

Cleft lip and palate (CLP) is a frequent congenital defect due to atypical orofacial development that displays racial and topographic differences.^1^ The worldwide prevalence of CLP is almost one in 700 live births, making it one of the most common congenital orofacial defects.^2^ The prevalence of CLP is also significantly higher in the Asian communities in comparison to other races.^3^ CLP patients come across multiple challenges, including aesthetic and functional problems.^4^ CLP is linked with a wide range of dental malformations that have serious long-term implications on the children’s facial profile and self-confidence.^5^ Dental abnormalities in CLP children may include enamel defects, hypodontia, supernumerary teeth, macrodontia, and microdontia.^6^ These abnormalities are reportedly more common in CLP children than in non-CLP children; and are more prevalent in permanent dentition than in primary dentition.^7^

Tooth agenesis of one or more permanent teeth without association with a systemic illness is common in CLP children.^7^ The incidence of congenitally missing teeth varies between 2.3% to 10.1%.^8^ The congenital missing several teeth (severe hypodontia) is usually associated with some syndromes 9. Severe hypodontia is commonly associated with hypohidrotic ectodermal dysplasia, incontinentia pigmenti, Down’s syndrome, and craniofacial dysostosis.^10^ Moreover, more than 60 syndromic conditions include dental agenesis as part of their phenotypic spectrum of anomalies associated with human mendelian inheritance.^11^ Missing teeth in the dentition have severe implications on speech, swallowing, dentition alignment, and psychological well-being, seriously affecting the quality of life in these individuals.^12^,^13^

The most commonly missing teeth in CLP children include maxillary lateral incisors, mandibular second premolars, and lower incisors.^2^ It is not clear if a similar pattern of tooth agenesis exists in Pakistani CLP children; and whether there is any difference in the pattern of congenitally missing teeth between the CLP and non-CLP Pakistani children.

Literature supports the genetic predisposition of tooth agenesis in CLP children.^14^ The associated factors contributing to the tooth agenesis inside or outside the cleft area are disruptions during development and iatrogenic injury during surgical interventions in the cleft region.^15^ In the initial phase of tooth development, surgical interventions are liable for tooth agenesis in the cleft region. In contrast, agenesis outside the cleft area is likely to be related to genetic predisposition.^16^ Information is scarce on the frequency of tooth agenesis in CLP Pakistani children. Previous studies conducted locally concluded that the tooth agenesis in cleft lip and plate were based on clinical examination and family history regardless of variation in different types of clefts.^17^ The objective of the present study was to establish the pattern of permanent tooth agenesis in CLP children in different classes using CBCT as a precision base line contemporary diagnostic aid in Lahore, Pakistan. Our hypothesis is to document the variation in tooth agenesis in cleft lip and palate cases.

## METHODS

A cross-sectional analytical study was conducted using Cone Beam Computed Tomography (CBCT) radiographs of CLP children who attended three radiology centers in Lahore, namely, Pakistan Jinnah MRI and Body Scan Center, Radiology Centers of the University of Lahore, and Fatima Memorial Hospital over a period of twelve months from September 2021 till August 2022. A retrospective evaluation was performed on CBCT images (Promax 3D, Romexis Software Version #5.1, Planmeca, Finland) across all three centers. Imaging protocol consistency lowers variability and potential biases in the collected data. Purposive (non-probability) sampling was employed. A sample size of 62 was estimated by using a 95% confidence level, 10% margin of error with expected agenesis prevalence on the non-cleft side as 20.9%.^15^ It was calculated by using the WHO calculator for prevalence studies.

### Ethical Approval:

This study was registered with the Office of Research, Innovation, and Commercialization (ORIC), and ethical approval was obtained from the IRB of the CMH Lahore Medical College & Institute of Dentistry, Lahore, Pakistan on 13^th^ Aug, 2021 (Letter No. 613/ERC/CMH/LMC) before the commencement of the study. All the collected information was anonymous, and no personal identifiers were recorded. Informed consent was obtained from the patient’s parents/legal guardians for participation in the study.

Healthy non-syndromic CLP patients of both genders aged 9-19 years were selected. The age bracket was chosen to ensure complete calcification of all permanent tooth crowns (which occurs at around 6-7 years of age). CLP diagnosis was confirmed through the medical records of the three selected centers. It was also ensured that no other congenital malformation or intellectual disorders were present in the chosen CLP children. The CBCT images taken according to the similar standardized procedure of the CLP centers were included in this study. The position and direction of patient during scanning follows the same standard procedure in three selected centers. The head of the patient will be positioned to bite the notched bite block and hold the machine with both arms in order to maintain a posture for CBCT imaging. Before CBCT scan calibration is a must followed procedure. Hence initially validation of CBCT is performed by using an object of known density (3D QA Phantom). The density of this phantom is close to realistic human tissues and geometry. The material of 3D QA Phantom provides the density of Tooth / Teeth which are represented in terms of acryl, aluminum and inside air. The densities of acryl, aluminum and inside air are provided by Planmeca Company. The details of density of each element of 3D QA Phantom can be given quality assurance test. The energy of the CBCT is set to 50keV-25 MeV.

Phantom is scanned with slice size 250 x 250 with increment 0.320 mm and pixel size 0.320 mm, FOV 8cm, gantry tilt 0.000 and number of slices 250. The energy of CBCT is set to 90.0kV 13.0mA 8.0s. For each CBCT scanning, the densities were measured through this quality assurance testing device software provided with Planmeca 3D Promax. The images with compromised quality (incomplete radiographic images or small Field of View (FOV), showing a limited number of teeth or medium FOV, and those showing only one arch were excluded from the study. The patients with a history of dental/jaw trauma, orthodontic treatment, and those under six years old were also excluded from the study. All radiographs were evaluated by the principal investigator (NZ) along with the second co-author (MS).

### Patterns of tooth agenesis:

Patients were classified into the following four categories: Cleft Lip and Palate Right (CLPR), Cleft Lip and Palate Left (CLPL), Bilateral Cleft (CLPB), and Midline Cleft (CLPM). The number of missing permanent teeth was counted in both maxillary and mandibular arches keeping in view the cleft group. Third molars were not included in the assessment. Tooth agenesis was scored as “1” and tooth present was scored as “0”.

### Statistical analysis:

The collected data were entered into a computer and analyzed using the Statistical Package for the Social Sciences (SPSS) Version #26.0 (IBM). Frequencies, means and standard deviations were generated for various variables. Two-way ANOVA was used to compare the means of the agenesis between the groups. Tukey’s test was used to determine where the differences were among the groups. The significance level was set at *p* ≤ 0.05. Intra- and inter-examiner reliabilities were measured by using Kappa statistics.

## RESULTS

The mean age of the selected 62 children was 14.2±4.4 years; 34 were male and 28 female. There was no difference in the results in terms of the gender of the CLP patients, so combined results are presented here. There were 22 children in the CLPR group, 26 in CLPL, 12 in CLPB and two in the CLPM group. The tooth agenesis prevalence was 69.4% among the sample. The highest agenesis cases (15 (24.2%)) were that of a single tooth. The overall agenesis and distribution among various CLP groups are presented in Table-I.

**Table-I:** Overall tooth agenesis and frequency distribution among various CLP groups.

Agenesis	Cleft Area Groups								Total	

	CLPR		CLPL		CLPB		CLPM			

	No.	%	No.	%	No.	%	No.	%	No.	%
None	9	40.9	8	30.8	2	16.7	0	0.0	19	30.6
1	3	13.6	11	42.3	1	8.3	0	0.0	15	24.2
2	8	36.4	3	11.5	5	41.7	0	0.0	16	25.8
3	0	0.0	3	11.5	1	8.3	0	0.0	4	6.5
4	2	9.1	1	3.8	1	8.3	1	50.0	5	8.1
5	0	0.0	0	0.0	0	0.0	1	50.0	1	1.6
7	0	0.0	0	0.0	1	8.3	0	0.0	1	1.6
8	0	0.0	0	0.0	1	8.3	0	0.0	1	1.6

Total	22	100	26	100	12	100	2	100	62	100

The mean tooth agenesis was highest (4.5 SD.71) in the CLPM group followed by CLPB (2.75 SD 2.49), CLPR (1.23 SD 1.27) and CLPL Group (1.15 SD 1.12) (Table-II). Several posterior teeth were found to be congenitally missing outside the cleft region, particularly in CLPB and CLPL groups. Tooth number five (permanent maxillary right 1^st^ premolar) was commonly missing outside the cleft region in all cleft types, followed by tooth #13 (permanent maxillary left 2^nd^ premolar). However, agenesis of other maxillary 1^st^ and 2^nd^ premolars were also observed compared to mandibular premolars (#20, #21, #28 and #29). The permanent mandibular lateral incisors (tooth #23 and #26) were found to be missing only in the unilateral CLPL group.

**Table-II:** Comparison of tooth agenesis between in-cleft and outside-cleft areas in relation to four CLP groups.

		Cleft_Area Groups				

		CLPR	CLPL	CLPB	CLPM	Total
Total Agenesis	Mean	1.23	1.15	2.75	4.5	1.6
	SD	1.27	1.12	2.49	0.71	1.69
	Minimum	0.0	0.0	0.0	4.0	0.0
	Median	1.0	1.0	2.0	4.5	1.0
	Maximum	4.0	4.0	8.0	5.0	8.0
Agenesis in posterior region	Mean	0.27	0.35	0.83	0.5	0.42
	SD	0.7	0.69	1.59	0.71	0.93
	Minimum	0.0	0.0	0.0	0.0	0.0
	Median	0.0	0.0	0.0	0.5	0.0
	Maximum	2.0	2.0	4.0	1.0	4.0
Agenesis in anterior region	Mean	0.95	0.81	1.92	4.0	1.18
	SD	1.13	0.63	1.16	0.0	1.14
	Minimum	0.0	0.0	0.0	4.0	0.0
	Median	0.5	1.0	2.0	4.0	1.0
	Maximum	4.0	2.0	4.0	4.0	4.0
In-cleft agenesis	Mean	0.86	0.73	1.58	4.0	1.05
	SD	1.08	0.53	0.9	0.0	1.03
	Minimum	0.0	0.0	0.0	4.0	0.0
	Median	0.5	1.0	2.0	4.0	1.0
	Maximum	4.0	2.0	3.0	4.0	4.0

**Fig.1:**
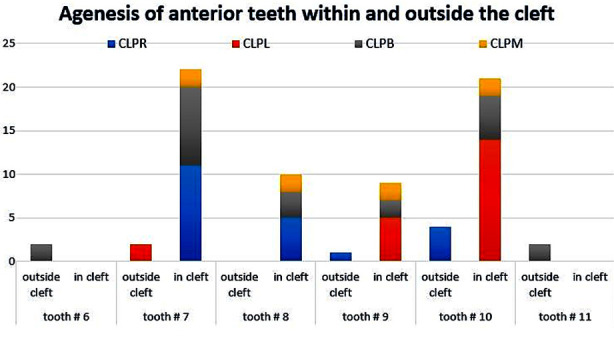
The Prevalence of anterior tooth agenesis: in-cleft and outside-cleft among maxillary and mandibular arches.

Agenesis of permanent anterior teeth was observed in all cleft types (Fig.1), with a higher prevalence among in-cleft areas. Permanent maxillary canines (#6 and #11) were missing in only two cases of bilateral cleft patients, but none in the in-cleft region. The bilateral cleft group showed a higher prevalence of permanent maxillary lateral incisors agenesis (#7 and 10) than central incisors (#8 and #9) in the in-cleft region. Furthermore, permanent maxillary right central and lateral incisors (#8 and #7) were commonly missing in the CLPR group. Similar was the case for agenesis of permanent maxillary left central and lateral incisors (#9 and #10) which were prevalent CLPL group. Two cases of midline cleft showed missing teeth number #7, #8, #9 and #10 inside the cleft (Fig.1).

When a comparison for overall agenesis was made among the four groups, the difference among groups was found significant with a *p*-value < 0.001. The group-wise comparisons revealed that CLPB and CLPM had significantly higher average agenesis than CLPR and CLPL, with *p*-values 0.030, 0.022, 0.017 and 0.017, respectively. The difference between CLPL and CLPR was insignificant with a *p*-value of 0.998 and between bilateral and midline with a *p*-value of 0.420 (Table-III).

**Table-III:** Overall and group-wise comparison for total agenesis among four groups by ANOVA followed by Tukey’s test.

Groups	CLPR	CLPL	CLPB	CLPM
Mean±SD	1.23±1.27 ^a^	1.15±1.12 ^ab^	2.75±2.49 ^c^	4.50±0.71 ^cd^
***From ANOVA *p*-value < 0.001***				

*(I) cleft area*	*(J) cleft area*	*Mean Difference (I-J)*	*Std. Error*	*p-value*

CLPR	CLPL	0.08	0.43	0.998
	CLPB	-1.52*	0.53	0.030
	CLPM	-3.27*	1.10	0.022
CLPL	CLPB	-1.60*	0.52	0.017
	CLPM	-3.35*	1.09	0.017
CLPB	CLPM	-1.75	1.13	0.420

* The difference is significant at 5% level of significance. The averages having no common superscript letter (a, b, c & d) have a significant difference.

## DISCUSSION

This study encompasses the prevalence and patterns of tooth agenesis among CLP patients in Lahore, Pakistan. The collected information would assist in the management and treatment planning for the CLP patients. As reported previously, the prevalence of the different cleft types and tooth agenesis did not vary significantly.^7^,^18^–^20^ The majority of the sample in this study belonged to the unilateral CLP groups with more missing teeth in the in-cleft area, again in agreement with other similar studies.^4^,^15^ However, agenesis was also observed in bilateral and midline cleft groups.

The present study showed that CLPL and CLPB groups commonly missing maxillary first premolars outside the cleft region compared to CLPM and CLPR groups. Furthermore, CLPL, CLPR, and CLPB type group cases showed missing second premolars outside the cleft region. This study depicts CLPL, CLPB, and CLPR groups showing a general pattern of maxillary premolars missing outside the cleft region. In a few cases, the CLPL group showed agenesis of mandibular lateral incisors in the mandibular arch.

Additionally, a negligible percentage of instances with missing mandibular first and second premolars were seen in the CLPR, CLPL, and CLPB group types. However, compared to mandibular premolars, maxillary premolars had a higher rate of agenesis. Contrary to our findings, earlier research reported more mandibular second premolars missing outside of the cleft.^18^,^19^ On the other hand, the prior study found that outside of the cleft, the CLPL group and alveolus region had more missing maxillary premolars and lateral incisors.^3^ These variations may result from the various populations under investigation, as racial characteristics influence the frequency of dental abnormalities. Genetic diversity within a community could cause the disparity in trends.^21^ However, all of these studies have in common the agenesis of premolars.

In the present study, two out of twelve CLPB patients showed missing maxillary right and left canine outside the cleft. Literature suggests scarce information regarding the pattern of missing canines in bilateral or other cleft types.^22^,^23^ A significant number of missing maxillary lateral incisors were observed inside the cleft in CLPR, CLPL and CLPB types. Few missing maxillary lateral incisors were seen outside CLPL and inside CLPM type cleft. A high number of cases also showed the absence of maxillary lateral incisor in CLPB type within the cleft. These findings are similar to the previous literature regarding the tooth number but dissimilarity due to more prevalence of agenesis outside cleft.^3^

The high prevalence of maxillary lateral incisors agenesis followed by maxillary second premolars agenesis may be attributed to failure of the union of maxillary and medial nasal processes. Previously very few cases of missing maxillary lateral incisors outside the cleft were observed.^2^,^24^ The difference in the findings might be due to the extent of malformation, severity, congenital predisposition, and absence of blood supply after the surgical procedure.

In the current study, only two midline cleft type cases were observed and both cases showed bilateral agenesis of maxillary central and lateral incisors within the cleft. Our findings are in agreement with the previous studies..^25^,^26^) To our knowledge, very little information on the pattern of missing teeth in midline cleft type is present until now.^15^ Moreover, maxillary central incisors are missing inside the cleft in CLPR, CLPL, CLPB and CLPM. Previous studies clearly explain that malformations show linkage with the type of missing teeth.22,27 The highest number of missing teeth were found in the midline cleft type. However, other cleft types also showed the absence of missing teeth, but to a lesser extent. These findings suggest the reason for the cleft-type malformations.

### Limitations:

It included the pattern of missing teeth in midline cleft type, which may not be considered for this region as a few cases were found in our study. The other limitation is that the data was localized and obtained from the diagnostic center in Lahore, and future study can be conducted on the national level to gather much greater data.

## CONCLUSION

Unilateral cleft lip and palate patients reported significant agenesis patttern compared to bilateral and median cleft cases. A multi-centered study with a higher sample size is recommended to validate these results further. The genetic aspect for the prevalence and type of tooth agenesis in specific cleft types also needs to be explored. Nevertheless, the present study has provided preliminary information about the prevalence of dental agenesis in cleft lip and palate children in Lahore, Pakistan.
